# Remote Ischemic Preconditioning Has No Short Term Effect on Blood Pressure, Heart Rate, and Arterial Stiffness in Healthy Young Adults

**DOI:** 10.3389/fphys.2019.01094

**Published:** 2019-08-21

**Authors:** Jan Müller, Marius Taebling, Renate Oberhoffer

**Affiliations:** Institute of Preventive Pediatrics, Technische Universität München, Munich, Germany

**Keywords:** arterial stiffness, remote ischemic preconditioning, arteriosclerosis – diagnosis, peripheral vascular, intervention

## Abstract

**Objective:**

Remote ischemic preconditioning (RIPC) are short episodes of ischemia and reperfusion applied to remote tissue to trigger responses in a specific organ or cardiovascular bed. This study investigates whether RIPC has a short-term effect on blood pressure (BP), heart rate, and arterial stiffness.

**Patients and Methods:**

From March 2018 to August 2018, we included 40 healthy volunteers (23 female, age 25.6 ± 2.8 years) into this single-blinded randomized-controlled crossover trial. After measuring BP, heart rate, and arterial stiffness in supine position participants were randomized into intervention or SHAM group. The intervention group then underwent a RIPC protocol (3 cycles of 5 min of 200 mmHg ischemia followed by 5 min reperfusion) at the thigh. The SHAM group followed the same protocol just on the upper arm with 40 mmHg pressure inflation. Directly after this 30-min procedure a reassessment of hemodynamic measures was conducted.

**Results:**

There were no significant changes in all five outcome parameters when comparing the effect of RIPC to SHAM. In peripheral systolic BP the mean difference between groups was Δ1.14 ± 6.5 mmHg (*p* = 0.672), and for diastolic BP Δ−0.69 ± 4.5 mmHg (*p* = 0.507). Heart rate shoed a Δ−0.8 ± 4.7 beats/min (*p* = 0.397). Regarding arterial stiffness measures, there was also no significant improvements thru RIPC. The mean difference between RIPC and SHAM for central systolic BP was Δ0.40 ± 7.2 mmHg (*p* = 0.951) and for PWV Δ0.01 ± 0.26 m/s (*p* = 0.563).

**Conclusion:**

This study could not find any short-term effects of RIPC on arterial stiffness, BP, and heart rate in a RCT in young healthy adults.

## Introduction

Steady oxygen supply is the basic requirement for humans to survive. However, intermittent hypoxic (Serebrovskaya, 2002) or ischemic (Heusch et al., 2015) conditions are often applied to trigger therapeutic adaptions in a variety of clinical diseases and emotional disorders. remote ischemic preconditioning (RIPC) are short episodes of non-fatal ischemia followed by reperfusion, usually applied to remote tissue for the purpose of protecting a specific organ or cardiovascular bed from future (preconditioning) or past (postconditioning) ischemic stimuli (Vasdekis et al., 2013; Heusch et al., 2015; Horiuchi, 2017). The signaling pathways are not completely understood so far but it involves neuronal, humoral, and systemic pathways (Hausenloy and Yellon, 2008; Hausenloy et al., 2015; Heusch et al., 2015; Anttila et al., 2016). RIPC can easily performed non-invasively in clinical practice by inflating and deflating a blood-pressure cuff on the upper arm or thigh, to induce transient ischemia, and reperfusion.

The idea of RIPC was originally to protect from myocardial injury during coronary artery bypass grafting first shown in 1993 (Przyklenk et al., 1993). In the subsequent years, many other experimental studies have also shown promising results (Hausenloy et al., 2007). Although a large randomized-controlled trial refuted previous findings recently (Hausenloy et al., 2015), there are many other protective effects of organs other than the heart reported for RIPC (Candilio et al., 2013). One significant is the endothelium, the inner cell layer of the vasculature which regulates vascular tone. Studies already pointed out that repeated RIPC improve endothelium-dependent vasodilation or protect endothelial function from ischemic injury (Loukogeorgakis et al., 2005; Kimura et al., 2007; Moro et al., 2011; Bailey et al., 2012; Jones et al., 2014, 2015; Epps et al., 2016). Since endothelial function regulates vascular tone, it is also a key component to determine arterial stiffness and blood pressure (BP). However, there are only few studies directly assessing BP and arterial stiffness in the context of RIPC bearing controversial findings. Kimura et al. (2007) found no altered BP after 4-weeks of daily RIPC even though endothelium-dependent vasodilation improved. In addition, Jones et al. (2015) could not find BP changes after 8-weeks of three times RIPC per week. Other small reports (Jones et al., 2014; Epps et al., 2016; Pryds et al., 2017) and case studies (Madias and Koulouridis, 2014; Madias, 2015) on the contrary found improved BP due to repeated RIPC exposure and there is only one study (Zagidullin et al., 2016) aiming at arterial stiffness and RIPC in patients with angina pectoris.

Overall, the short-term effect of RIPC on BP, heart rate, and arterial stiffness is unknown. Therefore, the aim of this study was to evaluate whether a single RIPC procedure shows a short-term improvement of BP and arterial stiffness.

## Patients and Methods

### Study Design and Subjects

From March 2018 to August 2018, we included 40 healthy volunteers without cardiovascular disease (23 female, age 25.6 ± 2.8 years, Table 1) into this single blinded randomized-controlled trial. Participants had no chronical diseases or acute infection during the study period. For standardization, all measurements were performed during 8:00 to 10:00 a.m. in the morning. The participants were free of infections, sober and did not consume any alcohol or tobacco for the last 12 h.

**TABLE 1:** Baseline characteristics of participants (*n* = 40).

**Anthropometrics**	
Gender (female)	23 (57.5%)
Age (years)	25.6 ± 2.8
Height (cm)	173.3 ± 9.7
Weight (kg)	65.5 ± 10.9
BMI (kg/m^2^)	21.7 ± 2.3
**Blood pressure and heart rate**	
Heart rate (beats/min)	66.5 ± 9.1
Systolic blood pressure (mmHg)	118.1 ± 8.8
Diastolic blood pressure (mmHg)	71.7 ± 6.5
**Arterial stiffness**	
Central systolic blood pressure (mmHg)	111.2 ± 9.9
Pulse wave velocity (m/s)	5.18 ± 0.37

After resting for 5 min in supine position the participants received a BP, heart rate, and arterial stiffness measurement on the left upper arm using oscillometric measurement device Mobil-O-Graph^®^ (IEM Healthcare, Stolberg Germany). Afterward they were randomized into an intervention or SHAM group using block method. The intervention group received a RIPC protocol of 3 cycles of 5 min each with occlusion to 200 mmHg with a special BP cuff on the right thigh followed by 5 min re-perfusion. The SHAM procedure consisted of a pressure cuff, inflated on the right upper arm for the same periods as the RIPC intervention but only to 40 mmHg as it has shown to mimic occlusion but not limiting blood flow. The study participants were thus led to believe that this study was a comparison between different occlusion techniques on the thigh and upper arm.

**FIGURE 1:**
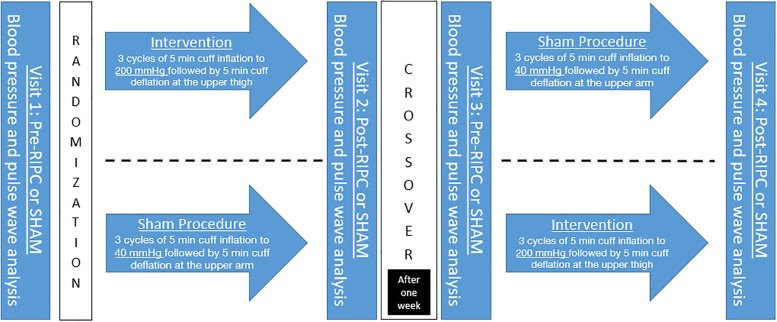
Study Protocol. RIPC, remote ischemic preconditioning.

Directly after this 30-min intervention or SHAM a reassessment of BP, heart rate and arterial stiffness measures was conducted. After a mean of 6.8 ± 1.0 days a crossover was performed and participants assigned to the other group and underwent the procedure again (Figure 1).

The study was conducted in accordance with the Declaration of Helsinki (revision 2008) and the Good Clinical Practice guidelines. The study protocol was approved by the local ethical board (project number 209/18S) of the Technical University Munich. All participants gave written informed consent and agreed to anonymous publication of their data.

### Blood Pressure, Heart Rate, and Arterial Stiffness Measurement

Blood pressure and arterial stiffness were automatically measured on the left upper arm with the oscillometric cuff-based Mobil-O-Graph device in a supine position after 5 min rest. Cuffs were chosen according to the circumference of the left upper arm (Elmenhorst et al., 2014; Meyer et al., 2017).

Central systolic blood pressure and pulse wave velocity (PWV) were indirectly estimated with an ARCSolver Algorithmus (Austrian Institute of Technology, Vienna, Austria) based on the recorded brachial pulse waves. This method includes the influence of arterial impedance and the aortic hemodynamics using a generalized transfer function and a mathematical model. It is confirmed that this non-invasive cuff-based method of the Mobil-O-Graph strongly correlates with the invasive measurement of central BP (Weber et al., 2011; Weiss et al., 2012; Townsend et al., 2015).

### Data Analyses

Data is described by mean ± standard deviation for all variables after proving normality for systolic, diastolic, central BP, and PWV by a Kolmogorov Smirnov test. To measure the effect of RIPC, differences between baseline and post-tests were calculated for intervention and SHAM procedures. Afterward the differences were compared with Student’s *t*-test for independent samples.

All tests were performed using SPSS (version 23.0, IBM Corporation). The level of significance for all two-tailed tests was set to <0.050.

## Results

There were no significant changes and very low effect sizes in all of the four outcome parameters when comparing the effect of RIPC to SHAM.

As shown in Table 2, in peripheral systolic BP the mean difference between groups was Δ1.14 ± 6.5 mmHg (effect size eta: 0.048; *p* = 0.672), and for diastolic BP Δ−0.69 ± 4.5 mmHg (effect size eta: 0.075; *p* = 0.507). There was also no significant change in heart rate (Δ−0.8 ± 4.7 beats/min; *p* = 0.397).

**TABLE 2:** Blood pressure and atrial stiffness parameters according to intervention and SHAM group.

	**Intervention group**			**SHAM group**			**Effect**		
	**Baseline**	**After intervention**	**Mean difference**	**Baseline**	**After SHAM procedure**	**Mean difference**	**Mean difference between groups**	**Eta**	***p*-value**
**Blood pressure**									
Heart rate (beats/min)	66.5 ± 9.1	60.6 ± 7.4	−5.9 ± 5.1	65.8 ± 8.3	60.7 ± 6.2	−5.1 ± 4.6	−0.8 ± 4.7	0.090	0.397
Systolic blood pressure (mmHg)	118.1 ± 8.7	119.5 ± 10.5	1.45 ± 7.1	117.9 ± 11.6	118.8 ± 11.0	0.83 ± 6.01	1.14 ± 6.5	0.048	0.672
Diastolic blood pressure (mmHg)	71.7 ± 6.5	70.7 ± 6.9	−1.03 ± 4.8	71.7 ± 7.2	71.4 ± 7.1	−0.35 ± 4.19	−0.69 ± 4.5	0.075	0.507
**Arterial stiffness**									
Central systolic blood pressure (mmHg)	111.2 ± 9.9	111.7 ± 10.5	0.45 ± 7.3	111.3 ± 10.5	111.6 ± 11.5	0.35 ± 7.26	0.40 ± 7.2	0.007	0.951
Pulse wave velocity (m/s)	5.18 ± 0.37	5.21 ± 0.41	0.03 ± 0.27	5.21 ± 0.43	5.20 ± 0.45	−0.01 ± 0.26	0.01 ± 0.26	0.066	0.563

Regarding arterial stiffness measures there were also no significant improvements through RIPC. The mean difference between RIPC and SHAM for central systolic BP was Δ0.40 ± 7.2 mmHg (eta: 0.007; *p* = 0.951) and for PWV Δ0.01 ± 0.26 m/s (eta: 0.066; *p* = 0.563).

Moreover, there were also no significant changes in systolic BP, diastolic BP, central systolic BP and PWV when comparing pre-RIPC with post-RIPC, and pre-SHAM with post-SHAM (Table 2, all *p* > 0.189). Only heart rate deceased in pre-RIPC with post-RIPC and pre-SHAM with post-SHAM significantly (both *p* < 0.001).

## Discussion

This study could not find any short-term effects of RIPC on BP, heart rate, and arterial stiffness in a randomized controlled trial with a crossover design in a big sample of 40 young healthy participants.

Remote ischemic preconditioning was originally designed to prevent the myocardium from subset ischemia during coronary artery bypass grafting (Przyklenk et al., 1993). There were studies showing promising results early on (Hausenloy et al., 2007), but bigger studies and randomized controlled trials have meanwhile cast doubt on the subject (Hausenloy et al., 2015). Nevertheless, there is compelling evidence measured mostly in the context of flow-mediated dilation that RIPC improves endothelial function (Kimura et al., 2007; Jones et al., 2014, 2015) and prevents the endothelial from injury during ischemia (Loukogeorgakis et al., 2005). Improvement or conservation of vascular function is a central feature because it reduces the risk for cardiovascular disease (Vita, 2011).

In addition to endothelial function, some studies assessed BP response to RIPC with controversial findings. In two different studies Jones et al. (2014, 2015) observed contradicting results in regard to different RIPC protocols. In the study with BP lowering effect they applied RIPC over seven consecutive days (Jones et al., 2014), whereas when using a protocol of just three times RIPC per week over a 8 week intervention period the BP lowering effect diminished (Jones et al., 2015). In the latter, they suggested that the lower stimuli have been of insufficient frequency to induce adaption in the cutaneous circulation. However, if the lower stimuli would be the reason in the second study (Jones et al., 2015), the study from Kimura et al. (2007) in which six single bouts of RIPC were performed daily over a period of 1 month should also have led to a drop in BP; but did not. The same is true for the study of Banks et al. (2016) where a 9-day RIPC period had also no effects. However, all the studies (Kimura et al., 2007; Jones et al., 2014, 2015; Banks et al., 2016) looked on normotensive and young participants and where BP lowering effects are hardly occur (floor effect). They also did not enable 24-h BP monitoring that would have brought more insights of the effect because RIPC has two phases; an early (up to 4 h) and late (after 24 h) (Loukogeorgakis et al., 2005). Especially on these two phases further studies should be aimed at in order to measure possible effects also on normotensive persons. Further it is still not clear which RIPC protocol is the most effective. Especially if one considers that a RIPC intervention with healthy vessels may have to be designed quite differently than in patients. Moreover. all of the studies including ours neglected hemodynamic conditions and loading conditions that have an important influence on BP. To mention at least heart rate that was similar pre SHAM and RIPC in our study and declined in parallel at post conditions which makes a possible bias unlikely.

On the other hand, in patients with chronic ischemic heart failure a 4-week RIPC program tended to improved systolic BP (Pryds et al., 2017) and it can therefore be assumed that the effect of RIPC is more pronounced when vascular health is already decreased. The same occurred in the study from Zagidullin et al. (2016) where the improved endothelial compliance and a reduction in peripheral systolic BP was outlined only in patients with angina pectoris although their RIPC protocol, which was similar to ours, consisted of only one RIPC of 3 × 5 min. Nevertheless the stimuli seem to be sufficient to trigger the humoral and neuronal mechanisms that mediate the endothelium by means of the sympathetic and parasympathtic nervous systems (Hausenloy and Yellon, 2008; Heusch et al., 2015; Zagidullin et al., 2016; Horiuchi, 2017). The merit of this study (Zagidullin et al., 2016) is that it is the only one which has investigated arterial stiffness using central systolic BP, which was also reduced after RIPC only in the angina pectoris group. Especially in hypertensive patients or patients with cardiovascular diseases, future studies must therefore be based on repeated RIPC exposure and focus on long-term assessment. Unfortunately, there are no other reports available that have investigated arterial stiffness measures like PWV, augmentation index or even the central BP in the context of RIPC. As endothelial function, arterial stiffness is also a subtle marker of arteriosclerosis. Therefore, changes in vascular tone mediated by the endothelium should come to light when assessing in arterial stiffness parameters (Laurent et al., 2006). In our healthy population, we assume that the single stimulus is not enough to trigger mechanisms that improve arterial stiffness. When we consider the relatively large number of cases in the present study then one can almost certainly say that a single RIPC has no short-term effect on BP and arterial stiffness in young healthy adults.

## Conclusion

This study could not find any short-term effects of RIPC on arterial stiffness and BP in a randomized controlled trial with a crossover design in a big sample of 40 young and healthy adults. Whether these findings also apply to patients with cardiovascular diseases must be clarified in further studies. Therefore, protocols have to be optimized with regard to duration and frequency of ischemia and reperfusion and the underlying mechanisms of RIPC have to be better understood (Heusch et al., 2015).

## Limitations

The study suffers from few volunteers and on higher number of cases should be aimed in further studies in order to take account of the high standard deviations in BP. Arterial stiffness and BP were recorded only once. For a more detailed BP analysis, multiple (favorable three) measures should be performed to determine BP, and arterial stiffness. However, it should be considered that multiple pre- and post- BP assessments are also short bouts of RIPC and could therefore bias the data. The use of our oscillometric device for the assessment of arterial stiffness utilizes cuff inflation to measure PWV and central pressure and thus adds an ischemic stimuli to the sham group and an additional ischemic stimuli to the intervention group. Devices using applanation tonometry would be more appropriate for this study.

## Data Availability

The datasets generated for this study are available on request to the corresponding author.

## Ethics Statement

Human Subject Research: The studies involving human participants were reviewed and approved by the Technical University Munich project number 209/18S. The patients and participants provided their written informed consent to participate in this study.

## Author Contributions

JM conceived and designed the study, sampled the parts of the analyzed data, and drafted the manuscript. MT sampled the data and provided important input for drafting and revising of the manuscript. RO conceived and designed the study and provided important input for revising of the manuscript.

## Conflict of Interest Statement

The authors declare that the research was conducted in the absence of any commercial or financial relationships that could be construed as a potential conflict of interest.
